# Antimicrobial susceptibility/resistance and genetic characteristics of *Neisseria gonorrhoeae* isolates from Poland, 2010-2012

**DOI:** 10.1186/1471-2334-14-65

**Published:** 2014-02-06

**Authors:** Beata Mlynarczyk-Bonikowska, Agnieszka Beata Serwin, Daniel Golparian, Szymon Walter de Walthoffen, Slawomir Majewski, Marta Koper, Magdalena Malejczyk, Marius Domeika, Magnus Unemo

**Affiliations:** 1 Department of Dermatology and Venereology, Medical University of Warsaw, Warsaw, Poland; 2 Department of Dermatology and Venereology, Medical University of Bialystok, Bialystok, Poland; 3 WHO Collaborating Centre for Gonorrhoea and other STIs, National Reference Laboratory for Pathogenic Neisseria, Department of Laboratory Medicine, Microbiology, Örebro University Hospital, Örebro, Sweden; 4 Department of Medical Microbiology, Medical University of Warsaw, Warsaw, Poland; 5 Department of Prevention and Control of Communicable Diseases, Uppsala County Council, Uppsala, Sweden

**Keywords:** *Neisseria gonorrhoeae*, Gonorrhoea, Poland, Antimicrobial resistance (AMR), Extended-spectrum cephalosporins (ESCs), Ceftriaxone, Cefixime, *penA*, NG-MAST

## Abstract

**Background:**

In Poland, gonorrhoea has been a mandatorily reported infection since 1948, however, the reported incidences are likely underestimated. No antimicrobial resistance (AMR) data for *Neisseria gonorrhoeae* has been internationally reported in nearly four decades, and data concerning genetic characteristics of *N. gonorrhoeae* are totally lacking. The aims of this study were to investigate the AMR to previously and currently recommended gonorrhoea treatment options, the main genetic resistance determinant (*penA*) for extended-spectrum cephalosporins (ESCs), and genotypic distribution of *N. gonorrhoeae* isolates in Poland in 2010-2012.

**Methods:**

*N. gonorrhoeae* isolates cultured in 2010 (n = 28), 2011 (n = 92) and 2012 (n = 108) in Warsaw and Bialystok, Poland, were examined using antimicrobial susceptibility testing (Etest), pyrosequencing of *penA* and *N. gonorrhoeae* multi-antigen sequence typing (NG-MAST).

**Results:**

The proportions of *N. gonorrhoeae* isolates showing resistance were as follows: ciprofloxacin 61%, tetracycline 43%, penicillin G 22%, and azithromycin 8.8%. No isolates resistant to ceftriaxone, cefixime or spectinomycin were found. However, the proportion of isolates with an ESC MIC = 0.125 mg/L, i.e. at the resistance breakpoint, increased significantly from none in 2010 to 9.3% and 19% in 2012 for ceftriaxone and cefixime, respectively. Furthermore, 3.1% of the isolates showed multidrug resistance, i.e., resistance to ciprofloxacin, penicillin G, azithromycin, and decreased susceptibility to cefixime (MIC = 0.125 mg/L). Seventy-six isolates (33%) possessed a *penA* mosaic allele and 14 isolates (6.1%) contained an A501V/T alteration in penicillin-binding protein 2. NG-MAST ST1407 (n = 58, 25% of isolates) was the most prevalent ST, which significantly increased from 2010 (n = 0) to 2012 (n = 46; 43%).

**Conclusions:**

In Poland, the diversified gonococcal population displayed a high resistance to most antimicrobials internationally previously recommended for gonorrhoea treatment and decreasing susceptibility to the currently recommended ESCs. The decreasing susceptibility to ESCs was mostly due to the introduction of the internationally spread multidrug-resistant NG-MAST ST1407 in 2011. It is essential to promptly revise the gonorrhoea treatment guidelines, improve the gonorrhoea laboratory diagnostics, and implement quality assured surveillance of gonococcal AMR (ideally also treatment failures) in Poland.

## Background

Infections caused by *Neisseria gonorrhoeae* are major public health problems globally. In 2008, the World Health Organization (WHO) estimated 106 million new cases of gonorrhoea among adults worldwide, which represents a 21% increase since 2005 [1]. In 2011, 39 179 gonorrhoea cases were reported from 28 European Union (EU)/European Economic Area (EEA) countries (data were not available from Germany and Liechtenstein), with an overall incidence of 12.6 cases per 100,000 population [2]. *N. gonorrhoeae* has developed antimicrobial resistance (AMR) to all drugs previously recommended for treatment of gonorrhoea. Extended-spectrum cephalosporins (ESCs) are the only remaining options for first-line empiric antimicrobial monotherapy in many countries worldwide [3-6]. However, verified treatment failures with particularly cefixime [7-13] but also rarely with ceftriaxone have been reported from several countries [14-18]. The first extensively-drug resistant (XDR) [3] gonococcal strains with high-level ESC resistance were also described recently [9,17,19]. In this worrying situation, the WHO [20], European Centre for Disease Prevention and Control (ECDC) [21] and Centers for Disease Control and Prevention (CDC), USA [22] have published action/response plans to mitigate the spread of multidrug-resistant gonorrhoea. A key component in these action/response plans is to substantially enhance the surveillance of gonococcal AMR worldwide. The European gonorrhoea treatment guideline was also revised in 2012, now recommending treatment with ceftriaxone 500 mg plus azithromycin 2 g [23]. Mutations in the *penA* gene (mosaic gene or A501 mutations) encoding the penicillin-binding protein 2 (PBP2) is the main determinant for decreased susceptibility and resistance to ESCs [5,9,17,24-30]. For molecular epidemiological typing of gonococci, the *N. gonorrhoeae* multiantigen sequence typing (NG-MAST) has been used in many countries [31].

**Figure 1:**
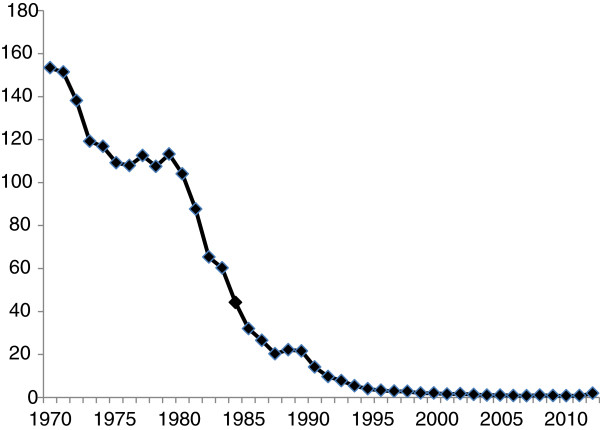
**Incidence of gonorrhoea in Poland in 1970-2012 (number of cases per 100,000 inhabitants).** Based on data from annual reports published by the National Institute of Public Health (http://www.pzh.gov.pl), the Former Institute of Venereology, and personal communication with Iwona Rudnicka, Chair and Department of Dermatology and Venereology, Medical University of Warsaw, Warsaw, Poland.

In the EU country Poland, gonorrhoea has been a mandatorily reported infection since 1948, when the incidence was approximately 180 cases per 100,000 inhabitants. Since 1970 (incidence of 153), the incidence has almost annually declined (with exception of from the mid- to late-1970s). The incidence of gonorrhoea from 1970 to 2012 in Poland is presented in Figure 1. Briefly, the incidence declined below 100 (87.6) in 1981, below 10 (9.7) in 1992, and below two in 2000. In 2012, the incidence was 1.9 (Figure 1). Data on the gonorrhoea incidence are available from annual reports; previously published by the former Institute of Venereology (currently, Chair and Department of Dermatology and Venereology, Warsaw, Poland), but since 1998 also by the National Institute of Public Health (http://www.pzh.gov.pl). Since 1974, procaine penicillin 4.8 million units intramuscularly together with the uriosuric drug probenecid 1.0 g orally have been recommended for treatment of gonorrhoea in Poland. Nevertheless, in practice a wide variety of antimicrobials are in use, i.e., particularly ceftriaxone (250-500 mg), but also cefixime, azithromycin, fluoroquinolones or tetracyclines [32,33]. A substantial proportion of cases are not diagnosed and treated by dermatovenereologists but, instead, by primary care physicians, urologists and gynaecologists, mainly private health care providers. In general, sensitive methods such as culture or nucleic acid amplification tests (NAATs) for detection of *N. gonorrhoeae* are rather rarely used in Poland. No AMR data for *N. gonorrhoeae* isolates from Poland, with exception of β-lactamase production, have been internationally reported since the 1970s [34-38]. Furthermore, no other phenotypic or genetic characteristics of gonococcal strains circulating in Poland have been published.

The aims of this study were to investigate the AMR to previously and currently recommended treatment options (to provide an evidence base for revision of the national treatment guidelines), the main genetic ESC resistance determinant (*penA*), and genotypic distribution of *N. gonorrhoeae* isolates in Poland in 2010-2012.

## Methods

### *Neisseria gonorrhoeae* isolates

*N. gonorrhoeae* isolates (n = 228) were obtained at the out-patient clinic of the Chair and Department of Dermatology and Venereology, Medical University of Warsaw, Poland (n = 218) and in two out-patients’ clinics in Bialystok, Poland (n = 10) in October 2010–December 2012. In total, 28 (in 2010), 92 (in 2011) and 108 (in 2012) isolates were collected. The isolates were cultured mainly from consecutive symptomatic gonorrhoea patients: 29 females and 199 males. Mean age for the females was 27 years (median age: 28 years; range: 17-40 years) and for the males 32 years (median age: 30 years; range: 17-74 years). One-hundred-eighty-eight (82.5%) isolates were obtained from specimens from male urethra, 28 (12.3%) from cervix, six (2.6%) from pharynx (from five males and one female) and six (2.6%) from male rectum.

All isolates were cultured on a modified Thayer-Martin selective agar media and subsequently confirmed as *N. gonorrhoeae* by identification of Gram negative diplococci in microscopy, rapid oxidase reaction, rapid sugar utilization test [39] and the PhadeBact GC Monoclonal test (Bactus AB, Solna, Sweden), and subsequently preserved as previously described [40]. All gonococcal isolates were cultured and stored as part of the routine diagnostics (standard care) and no patient identification information was used in the study. The isolates were later shipped on dry ice to the WHO Collaborating Centre for Gonorrhoea and other STIs, Sweden for further analysis. The 2008 WHO *N. gonorrhoeae* reference strains [41] were used for quality control of all phenotypic and molecular characterisation.

### Antimicrobial susceptibility testing

The minimum inhibitory concentration (MIC; mg/L) of cefixime, ceftriaxone, penicillin G, azithromycin, ciprofloxacin, spectinomycin, tetracycline and gentamicin were analysed using the Etest method on Difco GC Medium Base agar supplemented with 1% Isovitalex (bioMerieux AB, Solna, Sweden), according to the instructions from the manufacturer. All results were interpreted using whole MIC dilutions and where available, breakpoints for susceptibility (S) and resistance (R) according to The European Committee on Antimicrobial Susceptibility Testing (EUCAST [http://www.eucast.org]) were used (Table 1). For gentamicin, no breakpoints are stated by any organization. β-lactamase production was identified with nitrocefin discs.

**Table 1:** **Antimicrobial susceptibility of 228 *Neisseria gonorrhoeae *** **isolates from Poland in 2010-2012**

**Antimicrobial (Breakpoints (mg/L))**	**Susceptible no. (%)**			**Intermediate susceptible no. (%)**			**Resistant no. (%)**		
	**2010**	**2011**	**2012**	**2010**	**2011**	**2012**	**2010**	**2011**	**2012**
Ceftriaxone (S ≤ 0.125, I = NA, R > 0.125)*	28 (100)	92 (100)	108 (100)	NA	NA	NA	0	0	0
Cefixime (S ≤ 0.125, I = NA, R > 0.125)*	28 (100)	92 (100)	108 (100)	NA	NA	NA	0	0	0
Spectinomycin (S ≤ 64, I = NA, R > 64)*	28 (100)	92 (100)	108 (100)	NA	NA	NA	0	0	0
Azithromycin (S ≤ 0.25, I = 0.5, R > 0.5)*	23 (82.1)	60 (65.2)	63 (58.3)	2 (7.1)	26 (28.3)	34 (31.5)	3 (10.7)	6 (6.5)	11 (10.2)
Penicillin G (S ≤ 0.064, I = 0.125-1.0, R > 1.0)*	3 (10.7)	7 (7.6)	3 (2.8)	17 (60.7)	72 (78.3)	75 (69.4)	8 (28.6)	13 (14.1)	30 (27.8)
Tetracycline (S ≤ 0.5, I = 1.0, R > 1.0)*	12 (42.9)	31 (33.7)	26 (24.1)	4 (14.3)	26 (28.3)	31 (28.7)	12 (42.9)	35 (38.0)	51 (47.2)
Ciprofloxacin (S ≤ 0.032, I = 0.064, R > 0.064)*	10 (35.7)	46 (50)	34 (31.5)	0	0	0	18 (64.3)	46 (50)	74 (68.5)
Gentamicin**	MIC range: 0.032-8 mg/L; MIC_50_: 4 mg/L; and MIC_90_: 4 mg/L								
β-lactamase production	2010: 6 (21.4%) ; 2011: 2 (2.2%); and 2012: 4 (3.7%)								

NA, not applicable.

*Breakpoints for susceptible (S **≤** x mg/L) and resistant (R **>** y mg/L) according to The European Committee on Antimicrobial Susceptibility Testing (EUCAST; http://www.eucast.org).

**Breakpoints not stated by any organization.

### Isolation of genomic DNA

Bacterial DNA was isolated in the robotized NorDiag Bullet instrument (NorDiag ASA Company, Oslo, Norway) using the BUGS’n BEADS™ STI-*fast* kit (NorDiag ASA Company, Oslo, Norway), according to the instructions from the manufacturer.

### Molecular epidemiological typing

*N. gonorrhoeae* multiantigen sequence typing (NG-MAST) was performed as previously described [41,42]. NG-MAST allele numbers of the more variable segments of *porB* and *tbpB*, and sequence types (STs) were assigned using the NG-MAST website (http://www.ng-mast.net).

### Sequencing of the main ESC resistance determinant (*penA*)

Pyrosequencing of parts of the *penA* gene was performed as previously described [43].

### Sequence alignments

Multiple-sequence alignments of nucleotide sequences and the deduced amino acid sequences were performed in the software BioEdit Sequence Alignment Editor version 7.0.9.0 with manual adjustment.

### Statistical analysis

Statistical analysis was performed using the Statistica software version 9.0 PL (StatSoft Corporation, Cracow, Poland). Z-test for comparison of proportions was used. The level of significance was set at *P* < 0.05.

## Results

### Antimicrobial susceptibility of *N. gonorrhoeae* isolates (n = 228) in Poland

The results of the antimicrobial susceptibility testing of all isolates are summarized in Table 1.

Briefly, the overall proportions of isolates with resistance were as follows: ciprofloxacin 60.5%, tetracycline 43.0%, penicillin G 22.4%, and azithromycin 8.8%. The susceptibility to all those antimicrobials declined from 2010 to 2012, which was also statistically significant for tetracycline (*P* < 0.05) and azithromycin (*P* < 0.05). No isolates resistant to ceftriaxone, cefixime or spectinomycin were identified, and the MICs of gentamicin were relatively low (Table 1). Seven (3.1%) of the isolates showed multidrug resistance, i.e., resistance to ciprofloxacin, penicillin G, azithromycin, and decreased susceptibility to cefixime (MIC = 0.125 mg/L). The overall number of beta-lactamase producing *N. gonorrhoeae* isolates was 12 (5.3%), and the proportion of those significantly declined from 2010 to 2012 (*P* < 0.05) (Table 1).

Despite that no isolates resistant to ceftriaxone or cefixime (MIC > 0.125 mg/L) were identified, the proportion of isolates with ESC MICs ≤ 0.016 mg/L decreased significantly, from 78.6% (n = 22 in 2010) to 36.1% (n = 39 in 2012) for ceftriaxone (*P* < 0.05) and from 89.3% (n = 25 in 2010) to 36.1% (n = 39 in 2012) for cefixime (*P* < 0.05). Furthermore, the proportion of isolates with an ESC MIC = 0.125 mg/L (the EUCAST breakpoint for resistance) increased significantly, from none in 2010, to one (1.1%) and seven (7.6%) in 2011, and to 10 (9.3%) and 21 (19.4%) in 2012 for ceftriaxone and cefixime, respectively (*P* < 0.05). Isolates with an ESC MIC of 0.125 mg/L have resulted in treatment failures with ESCs [13,15,16,44] and, accordingly, can be considered as having at least a decreased susceptibility to ESCs.

### ESC resistance determinant (*penA*) and molecular epidemiology

Overall, 76 (33.3%) isolates contained a *penA* mosaic allele, and the MICs of ceftriaxone and cefixime for these isolates ranged from 0.002-0.125 mg/L (mean MIC: 0.032 mg/L) and <0.016 to 0.125 mg/L (mean MIC: 0.064 mg/L), respectively. Thirteen isolates (5.7%) contained a PBP2 A501V alteration and one (0.4%) a PBP2 A501T alteration, and the MICs of ceftriaxone and cefixime for these isolates ranged from 0.016 to 0.125 mg/L (mean MIC: 0.064 mg/L) and from <0.016 to 0.064 mg/L (mean MIC: 0.032 mg/L), respectively. The NG-MAST STs, MICs of ESCs, and presence of the main ESC resistance determinants (*penA* mosaic allele and PBP2 A501-altered allele) in all isolates are summarized in Table 2.

**Table 2:** ***Neisseria gonorrhoeae *** **multiantigen sequence typing (NG-MAST) STs, minimum inhibitory concentrations (MICs, mg/L) of cefixime and ceftriaxone, and *penA *** **alterations in *Neisseria gonorrhoeae *** **(n = 228) isolated in Poland, in 2010-2012**

**NG-MAST ST (No. of isolates)**	**Drug**	**No. of isolates with MIC (mg/L):**					***penA***
							**alteration**^***a***^
		≤**0.016**	**0.032**	**0.064**	**0.125**	**0.25**	
1407 (58)	IX		4	30	24		58 (100%) mosaic
	TX	4	18	27	9		
2992 (17)	IX	17					17 (100%) WT
	TX	17					
1405 (12)	IX	3	8	1			12 (100%) A501V
	TX	2	6	4			
1861 (10)	IX	5	5				10 (100%) WT
	TX	1	4	5			
8392 (8)^*b*^	IX	8					8 (100%) WT
	TX	8					
8379 (8)^*b*^	IX	8					8 (100%) WT
	TX	6	2				
8391 (7)^*b*^	IX	7					7 (100%) WT
	TX	7					
5421 (7)	IX	7					7 (100%) WT
	TX	7					
8370 (6)	IX	6					6 (100%) WT
	TX	6					
1478 (5)	IX	5					5 (100%) WT
	TX	5					
7674 (5)	IX	5					5 (100%) WT
	TX	5					
21 (5)	IX	4	1				5 (100%) WT
	TX	2	1	2			
STs with 2-4 isolates (42)^*c*^	IX	30	6	4	2		8 (19%) mosaic; 34 (81%) WT
	TX	31	6	5			
Unique STs (38)^*d*^	IX	28	3	5	2		10 (26%) mosaic; 1 (2.6%) A501V; 1 (2.6%) A501T; 26 (68%) WT
	TX	28	4	4	2		

NG-MAST, *Neisseria gonorrhoeae* multiantigen sequence typing; ST, sequence type; MIC, minimum inhibitory concentration; IX, cefixime; TX, ceftriaxone; WT, wild type.

^*a*^Presence of *penA* mosaic allele or alteration of amino acid A501 in the penicillin-binding protein 2, which both have been associated with decreased susceptibility to extended-spectrum cephalosporins [5].

^*b*^Novel sequence types found in the present study.

^*c*^STs represented by 2-4 isolates; 225 (4), 8387 (4)^*b*^, 8373 (3)^*b*^, 298 (3), 1340 (3), 8385 (3)^*b*^, 4758 (3), 735 (2), 2226 (2), 5004 (2), 8377 (2)^*b*^, 8378 (2)^*b*^, 8393 (2)^*b*^, 8394 (2)^*b*^, and 8399 (2)^*b*^.

^*d*^Unique STs represented by single isolates.

Briefly, twenty-eight (93.3%) of the 30 isolates with decreased susceptibility (MIC = 0.125 mg/L) to ceftriaxone (n = 2), cefixime (n = 19) or both ESCs (n = 9) contained a *penA* mosaic allele, and one (3.3%) contained an A501V alteration in PBP2. Furthermore, 25 (83.3%) of these 30 isolates belonged to NG-MAST ST1407.

In total, the 228 isolates were assigned to 66 different NG-MAST STs. Thirty-four (51.5%) of these STs were not previously described. The most prevalent ST was ST1407 (25.4% of isolates), followed by ST2992 (7.5%), ST1405 (5.3%), ST1861 (4.4%), ST8392 (3.5%), and ST8379 (3.5%) (Table 2). The proportion of isolates of the most prevalent ST (ST1407) rapidly and significantly increased from none in 2010, to 12 (13.0%) in 2011 and finally to 46 (42.6%) in 2012 (*P* < 0.05). All ST1407 isolates (100%) contained a mosaic *penA* allele (Table 2).

## Discussion

The present study describes the first internationally reported gonococcal AMR data in nearly four decades and the first molecular characterization of *N. gonorrhoeae* isolates in Poland. Despite the global concern of multidrug-resistant and possibly future untreatable gonorrhoea [5], the last published AMR data, with exception of β-lactamase production [36,37], for gonococcal isolates from Poland are from the mid-to late-1970s and only published in Polish [34,35,38]. Furthermore, the susceptibility to internationally frequently used antimicrobials such as ceftriaxone, cefixime, spectinomycin, ciprofloxacin, and azithromycin has never been assessed. Overall, high prevalence of resistance, including multidrug resistance, was observed for internationally previously recommended antimicrobials such as ciprofloxacin (60.5%), tetracycline (43.0%), and penicillin G (22.4%), and the latter remains nationally recommended for treatment of gonorrhoea in Poland [32,33]. Five per cent (range: 2.2%-21.4% during 2010-2012) of the gonococcal isolates in Poland were β-lactamase producing, which is substantially higher than the previously reported prevalence of 0.8% (range: 0-1.1%) during 2006-2009 [37]. The prevalence of resistance to azithromycin was also relatively high (8.8%). Similar levels of resistance to ciprofloxacin, tetracycline, penicillin G and azithromycin have been described from many other countries in Europe and basically worldwide [3-6,20-22,45,46], and none of these antimicrobials should be recommended for empiric first-line antimicrobial monotherapy in Poland or in many other countries globally. Accordingly, it is essential to promptly revise and strictly implement an up-to-date treatment guideline for gonorrhoea in Poland. The results of the present study provide a strong evidence base for this revision of the Polish treatment guidelines, which has been initiated. No isolates with resistance (MIC > 0.125 mg/L) to ceftriaxone or cefixime were found. However, worryingly the proportion of isolates displaying decreased susceptibility to ESCs (MIC = 0.125 mg/L), which have previously resulted in gonorrhoea ESC treatment failures [13,15,16,44], rapidly increased from none in 2010, to 7.6% in 2011, and 13.9% in 2012. Ninety-three per cent of these isolates contained a mosaic *penA* allele, which has been associated with decreased susceptibility or resistance to ESCs in many countries [3,5,9-19,24-30]. It is crucial to continuously follow the spread of gonococcal strains with multidrug resistance and decreased susceptibility or future resistance to ESCs in Poland. Accordingly, gonococcal AMR (ideally also treatment failures) will be surveyed annually in Poland (isolates are prospectively collected) and preparations for joining the European Gonococcal Antimicrobial Surveillance Programme (Euro-GASP) [46,47], which acts in the EU/EEA, have been initiated.

Using NG-MAST, the present study identified a diversified gonococcal population in Poland during 2010-2012, with 66 different NG-MAST STs among the 228 isolates. ST1407 (25.4% of all isolates) was the most frequent ST and also significantly increased from 2010 (0%) to 2012 (42.6%). Of the ST1407 isolates, 15.5% displayed decreased susceptibility to ceftriaxone and 41.4% to cefixime, and all contained a mosaic *penA* allele. NG-MAST ST1407 has been previously shown to account for most of the decreased susceptibility or resistance to ESCs in Europe and to be responsible for treatment failures with cefixime in Norway [11], Austria [10], France [9] and Canada [13], as well as with ceftriaxone in Slovenia [16]. The high number of unique STs (n = 38) and novel STs (n = 34), which were identified in Poland, may be associated with suboptimal diagnostics (only random gonorrhoea patients and/or isolates are identified), contact tracing (sexual contacts having the identical ST are not traced) and epidemiological surveillance (sexual transmission chains spreading a single ST are not identified or followed-up), locally evolved STs (novel STs because no NG-MAST studies have been previously performed in the country) or STs imported from abroad. However, some main ST clusters caused by clonal spread of, e.g., ST1407 (n = 58), ST 2992 (n = 17), ST1405 (n = 12), and ST1861 (n = 10) were identified, which indicate some larger sexual transmission chains.

The reported incidences of gonorrhoea in Poland (<2 cases per 100,000 inhabitants annually during last decade) have been substantially lower than in neighbouring countries/regions, for example, in 2011 in Belarus (33.4), Kaliningrad Oblast (27.5), Ukraine (20.1), Lithuania (6.7), Czech Republic (6.7) and Slovakia (3.6) [2,47]. The gonorrhoea incidence in Poland is likely underestimated, due to suboptimal diagnostics (more sensitive methods such as NAATs and/or culture need to be more frequently used), access to appropriate diagnostics and lack of screening of asymptomatic patients, and incomplete case reporting (particularly among private health care providers) as well as epidemiological surveillance.

## Conclusion

*N. gonorrhoeae* isolates cultured in Poland in 2010-2012 showed a high genetic diversity and high levels of resistance to antimicrobials internationally earlier recommended for gonorrhoea treatment, e.g., ciprofloxacin, tetracycline, and penicillin G. No resistance, however, relatively high and rapidly increasing levels of decreased susceptibility (MIC = 0.125 mg/L) to ceftriaxone and to cefixime were identified, which was mostly due to the introduction of the internationally spread multidrug-resistant NG-MAST ST1407 in Poland in 2011. It is essential to promptly revise the gonorrhoea treatment guidelines (which the present study provides an evidence base for), improve the gonorrhoea laboratory diagnostics, and implement quality assured surveillance of gonococcal AMR (ideally also treatment failures) in Poland.

## Competing interests

The authors declare that they have no competing interests.

## Authors’ contributions

MU, MD, BM-B and ABS designed the study. BM-B, ABS, DG, SWW, SM, MK and MM collected the isolates and approved the study design. DG, BM-B, MK and SWW performed all the laboratory analysis. BM-B, ABS, DG and MU analysed and interpreted all the data, and wrote a first draft of the paper. All authors read, commented on and approved the final manuscript.

## Pre-publication history

The pre-publication history for this paper can be accessed here:

http://www.biomedcentral.com/1471-2334/14/65/prepub
